# Genotyping of human neutrophil antigens (HNA) from whole genome sequencing data

**DOI:** 10.1186/1755-8794-6-31

**Published:** 2013-09-12

**Authors:** Hsueh-Ting Chu, Han Lin, Theresa Tsun-Hui Tsao, Chun-Fan Chang, William WL Hsiao, Tze-Jung Yeh, Ching-Mao Chang, Yen-Wenn Liu, Tse-Yi Wang, Ko-Chun Yang, Tsung-Jui Chen, Jen-Chih Chen, Kuang-Chi Chen, Cheng-Yan Kao

**Affiliations:** 1 Department of Biomedical informatics, Asia University, Taichung 41354, Taiwan; 2 Department of Computer Science and Information Engineering, Asia University, Taichung 41354, Taiwan; 3 Department of Computer Science and Information Engineering, National Taiwan University, Taipei 10617, Taiwan; 4 Graduate Institute of Biotechnology, Chinese Culture University, Taipei 11114, Taiwan; 5 Department of Pathology and Laboratory Medicine, University of British Columbia, Vancouver, BC V5Z 4R4, Canada; 6 Institute of Biotechnology, National Taiwan University, Taipei 10617, Taiwan; 7 Graduate Institute of Clinical Medical Science, Chang Gung University, Taoyuan 33302, Taiwan; 8 National Research Institute of Chinese Medicine, No. 155-1, Section 2, Li-Nong StreetBeitou District, Taipei 11221, Taiwan; 9 Laboratory of Molecular Anthropology and Transfusion Medicine, Mackay Memorial Hospital, New Taipei City 25160, Taiwan; 10 Department of Medical Informatics, Tzu Chi University, Hualien 97004, Taiwan

**Keywords:** Antigens, Neutrophil, Genotyping, Whole genome sequencing

## Abstract

**Background:**

Neutrophil antigens are involved in a variety of clinical conditions including transfusion-related acute lung injury (TRALI) and other transfusion-related diseases. Recently, there are five characterized groups of human neutrophil antigen (HNA) systems, the HNA1 to 5. Characterization of all neutrophil antigens from whole genome sequencing (WGS) data may be accomplished for revealing complete genotyping formats of neutrophil antigens collectively at genome level with molecular variations which may respectively be revealed with available genotyping techniques for neutrophil antigens conventionally.

**Results:**

We developed a computing method for the genotyping of human neutrophil antigens. Six samples from two families, available from the 1000 Genomes projects, were used for a HNA typing test. There are 500 ~ 3000 reads per sample filtered from the adopted human WGS datasets in order for identifying single nucleotide polymorphisms (SNPs) of neutrophil antigens. The visualization of read alignment shows that the yield reads from WGS dataset are enough to cover all of the SNP loci for the antigen system: HNA1, HNA3, HNA4 and HNA5. Consequently, our implemented Bioinformatics tool successfully revealed HNA types on all of the six samples including sequence-based typing (SBT) as well as PCR sequence-specific oligonucleotide probes (SSOP), PCR sequence-specific primers (SSP) and PCR restriction fragment length polymorphism (RFLP) along with parentage possibility.

**Conclusions:**

The next-generation sequencing technology strives to deliver affordable and non-biased sequencing results, hence the complete genotyping formats of HNA may be reported collectively from mining the output data of WGS. The study shows the feasibility of HNA genotyping through new WGS technologies. Our proposed algorithmic methodology is implemented in a HNATyping software package with user’s guide available to the public at http://sourceforge.net/projects/hnatyping/.

## Background

Transfusion-related acute lung injury (TRALI) is a serious cause of transfusion-related morbidity and mortality [1,2]. Published evidence strongly suggests that antibodies against neutrophil antigens, including the 7 antigens HNA-1a, HNA-1b, HNA-1c, HNA-2, HNA-3a, HNA-4a and HNA-5a, have all been implicated in cases of TRALI [3]. These antibodies lead to the activation of neutrophil that induces endothelial and alveolar damage in the lungs [2,4,5]. These antigens have also been reported to cause alloimmune neonatal neutropenia (ANN) [6].

The HNA-1 antigens are located on the low-affinity Fc-γ receptor IIIb (FCGR3B), CD16b. These receptors bind to the Fc portion of IgG antibodies [7]. The HNA-2 antigen system is located on CD177. The number of neutrophils expressing HNA-2 may increase in pregnancy, infections and myeloproliferative disorders [8]. The HNA-3 antigen system has one antigen, HNA-3a. HNA-3a is expressed by neutrophils, lymphocytes, platelets, endothelial cells, kidney, spleen, and placental cells. The antigen is located in exon 7 of the CLT2 gene (SLC44A2) [9]. The HNA-4 and HNA-5 antigens are located in the β2 integrins. Each antigen system contains only a single antigen. HNA-4a has been located on the αM chain (CD11b) [10]. HNA-5a has been located on the αL integrin unit (CD11a) [10]. Table 1 lists the five antigen systems and their responding genes.

**Table 1:** Current nomenclature for human neutrophil antigens and corresponding genes

**System**	**Antigens**	**Gene**	**cDNA sequence**	**Nucleotides (dbSNP ID)**
	HNA-1a,	FCGR3B	NM_000570.4	141(G),
HNA-1	HNA-1b	FCGR3B	NM_000570.4	141(C), 266(C)
	HNA-1c	FCGR3B	NM_000570.4	141(C), 266(A)
HNA-2	HNA-2a	CD177		
HNA-3	HNA-3a	SLC44A2	NM_020428.3	600(G) (rs2288904)
	HNA-3b	SLC44A2	NM_020428.3	600(A)
HNA-4	HNA-4a	ITGAM	NM_001145808.1	327(G) (rs17362505)
	HNA-4b	ITGAM	NM_001145808.1	327(A)
HNA-5	HNA-5a	ITGAL	NM_001114380.1	2296(G) (rs2230433)
	HNA-5b	ITGAL	NM_001114380.1	2296(C)

With the invention of polymerase chain reaction (PCR), different PCR-based genotyping assays on HNA have been developed, including sequence-based typing (SBT) [11] as well as PCR-sequence-specific oligonucleotide probes (SSOP) [12], PCR-sequence-specific primers (SSP) [13] and PCR-restriction fragment length polymorphism (RFLP) [14]. Sequence-specific oligonucleotide probes (SSOP) and sequence-specific primers (SSP) were designed to detect variable sequence motifs in PCR-amplified HNA genes, revealing an extensive level of previously detected alleles. Sequence-based typing (SBT) enables all nucleotides to be identified so that it provides a better method for the evaluation of new alleles on HNA genes. PCR-based methods continue to be used in routine antigen detection. However, the next-generation sequencing technology (NGS) enables rapid whole-genome sequencing for more detailed and precise investigation of total variants of genes [15]. It opens the opportunity to redesign genotyping strategies for more effective genetic mapping and genome analysis [16].

In this paper, we developed a bioinformatics tool for typing the HNA antigens from personal human whole-genome sequencing data. The NGS technology was evaluated for its potential in high-resolution HNA typing. Our tool combined with NGS data can produce unambiguous results regardless any new identified variants of the antigen systems.

## Implementation

**Figure 1:**
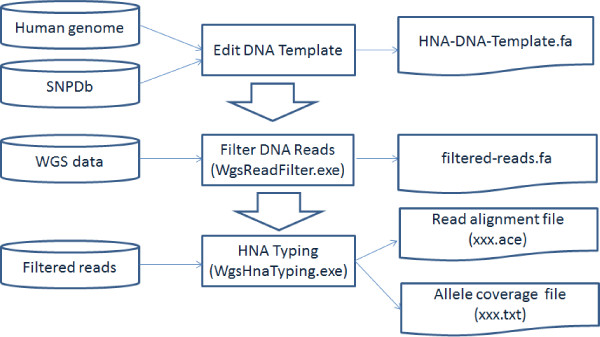
**Overview of WGS-based HNA typing.** The HNA-DNA-template and the programs WgsReadFilter and WgsHnaTyping are available from the supplemental software of this paper.

Human whole-genome sequencing with next-generation sequencing usually produces hundreds of gigabases, thus WGS mapping or assembly tools generally requires large memory such that it is intractable to use such tools for specific genotyping. In this study, we developed a light-weight method for the purpose of HNA typing as shown in Figure 1. There are three steps in our genotyping procedure. Firstly, the procedure specifies a set of short DNA sequences (~200bp) which contain all of the nucleotide variants of antigens. Secondly, the DNA sequences are used to filter WGS datasets. These DNA sequences are only 1/100000 of the human whole genome such that most of the unrelated reads in WGS dataset are removed and only a small set of reads are kept for typing. The final step is the alignment of the filtered reads on to the DNA template. We developed two programs, WgsReadFilter and WgsHnaTyping, for the procedure. These programs only consume small amount of memory to deal with the large WGS datasets. The detail of the procedure is described as following.

### The identification of DNA variants for the HNA systems

The nucleotide polymorphisms for recognition of different HNA antigens have been published in literature [3,17,18] (Table 1). But the previously reported nucleotide polymorphisms are not consistent. For example, the allele for HNA5a was reported to be at ITGAL*2372 by Moritz [3] or at ITGAL*2466 by Xia [18], furthermore, both of these loci are inconsistent with the GenBank sequences (NM_001145056.1 or NM_002209.2) of human ITGAL genes. Consequently, we searched the Single Nucleotide Polymorphism database (dbSNP) to ensure the correctness of the loci for HNA alleles. For the HNA-1 system, there are two sites, 141 and 266, to discriminate HNA-1a, -1b, and -1c antigens. Variants for HNA-2 had not been revealed. Each of the other HNA systems can be recognized with a single nucleotide polymorphism (SNP). Our curated alleles for HNA-3a, -4a, and -5a are identified by the SNP IDs, rs2288904 (HNA3), rs1143679 (HNA4) and rs59554592 (HNA5) and the loci of these SNPs are listed in Table 1. The loci of these SNPs in the human genome (GRCh37.p5) are listed in Table 2. We downloaded +/−100 bp of flanking sequence surrounding the SNPs for all alleles, which is used as DNA templates for filtering and alignment of reads.

**Table 2:** DNA variants in human genome for the HNA antigens

**SNPs**	**Chromosome**	**Locus (GRCh37.p5)**	**Oligonucleotide (21mer) on the SNP**
FCGR3B*141G/C	Chr. 1	13,007,064	AATGGTACAG[C/G]GTGCTCGAGA
FCGR3B*266C/A	Chr. 1	13,006,939	TTCATTGACG[C/A]TGCCACAGTC
CTL2*461A/G	Chr. 9	2,004,972	GAGGTGCTTC[A/G]AGATGGTGAC
ITGAM*230A/G	Chr.19	31,216,811	GAGCCCATCC[A/G]CCTGCAGGGT
ITGAL*2372C/G	Chr.16	30,458,041	CACAGATCCA[C/G]AGCCCTGCGT

### The filtering of HNA reads from WGS datasets

To filter the reads, we build a hash table of short keys (default is 14 bp) from the DNA templates stated in the previous section. We examined the non-overlapped occurrences of the keys on each read and the reads with more than two occurrences of keys are stored for the typing. The filtering process is used to efficiently eliminate most of the unrelated reads to speed up the following alignment process. Only very few reads remain in the output of this step.

### The typing of HNA reads by the alignment of the filtered reads

**Figure 2:**
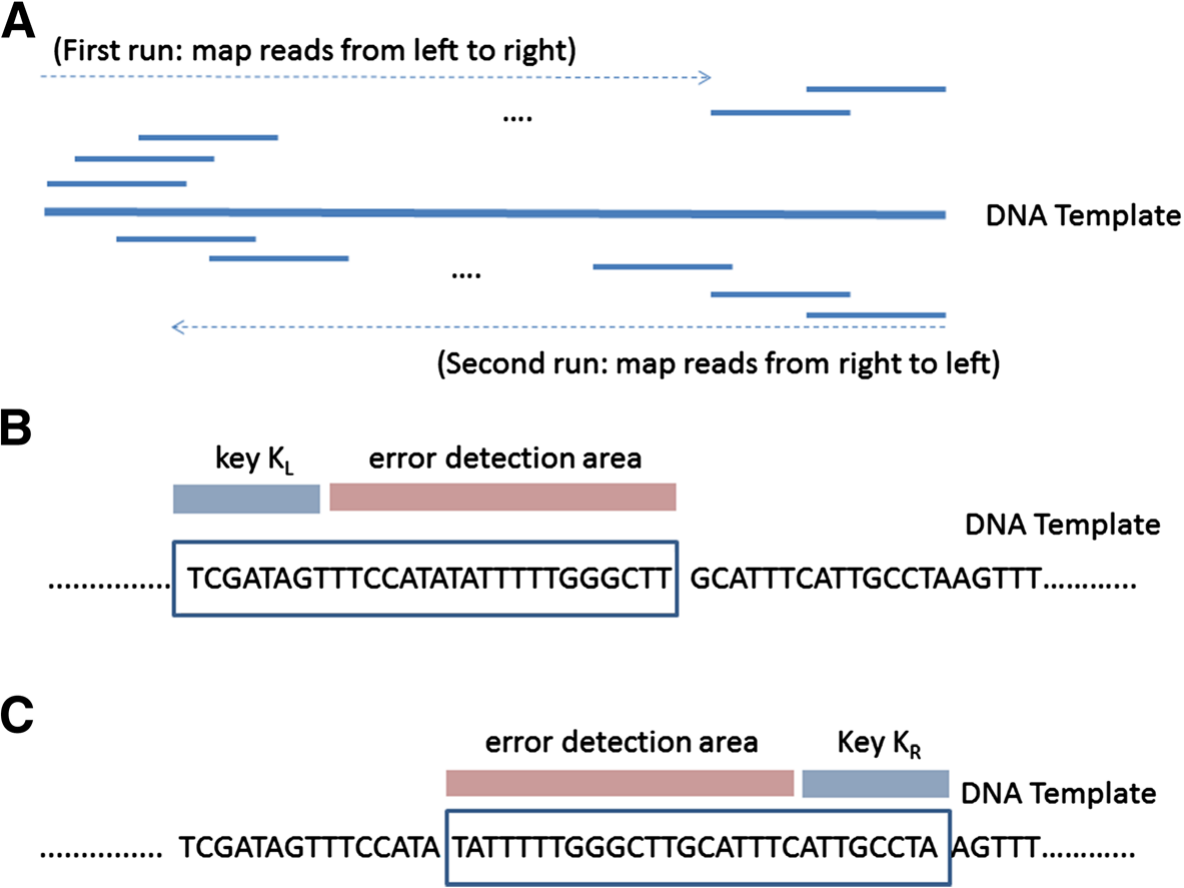
**The mapping algorithm of WgsHnaTyping. A)** The mapping algorithm searches twice for candidates reads. First time, the searching is performed from the 5′ end to the 3 end and then again from right to left. **B)** For mapping from the 5′ end, the prefixes of reads are used as the index key. **C)** For mapping from the 3′ end, the postfixes of reads are used as the index key.

The filtered reads were mapped onto the DNA templates in the WgsHnaTyping program. As shown in Figure 2, the mapping procedure executed twice for each DNA template. First, the mapping was performed from the 5′ end to the 3′ end of the template sequence, and then it was performed again from the 3′ end to the 5′ end (Figure 2A). Then the candidate reads were screened by index keys K_L_ and K_R_. The prefixes of reads were used as the index key K_L_ when the mapping is from the 5′ end (Figure 2B). Similarly, the postfixes of reads were used as the index key K_L_ when the mapping is from the 3′ end (Figure 2C). The default key length was 15 bp. Moreover, the filtered reads were compared with the template sequence by the error detection area. Finally, those reads were aligned if there were less than 1/10 error bases.

Genotype calling would then proceed by counting the number of times each allele is observed and using a fixed cut off value. For example, a heterozygous genotype is called if the proportion of the non-reference allele is between 5% and 95%; otherwise, a homozygous genotype would be called. But if the coverage at the SNP site is less than 2, the “unknown” genotype would be called. The WgsHnaTyping program displays the results of HNA types in a user friendly graphical user interface and also outputs the following two files - (1) an ACE file which recorded the alignment of reads. The ace file can be displayed with the visualization tool UGENE (http://ugene.unipro.ru/), and (2) a TXT file to record the coverage at each allele.

### Whole-genome sequencing samples

Six public WGS datasets were downloaded from the European Nucleotide Archive (http://www.ebi.ac.uk/ena/). Table 3 lists the six samples. The WGS datasets were from two pedigree trios: YOR009 and CEPH146 of the 1000Genomes project [19]. YOR009 is an African family. CEPH1463 is an American family from Utah with Northern and Western European ancestry. These DNA samples were isolated from B-lymphocyte cells derived from blood.

**Table 3:** Whole genome sequencing samples

**HapMap family**	**Sample**	**Sex**	**Relation**	**SRA ID of dataset**^**a,b**^	**Total reads in dataset**	**Filtered reads for HNA typing**
YOR009	NA18507	male	father	ERX009609	1340M	3,542
	NA18508	female	mother	ERX009610	1327M	3,313
	NA18506	male	child	ERX009608	1320M	3,384
CEPH146	NA12891	male	father	ERX000172 ~ 3	980M	678
	NA12892	female	mother	ERX000174 ~ 5	978M	566
	NA12878	female	child	ERX000170 ~ 1	1109M	656

^a^The length of reads in the experiments ERX009608 ~ ERX009610 is 100bp from Illumina Genome Analyzer II.

^b^The length of reads in the experiments ERX000170 ~ ERX000175 is 36bp from Illumina Genome Analyzer.

## Results and discussion

**Figure 3:**
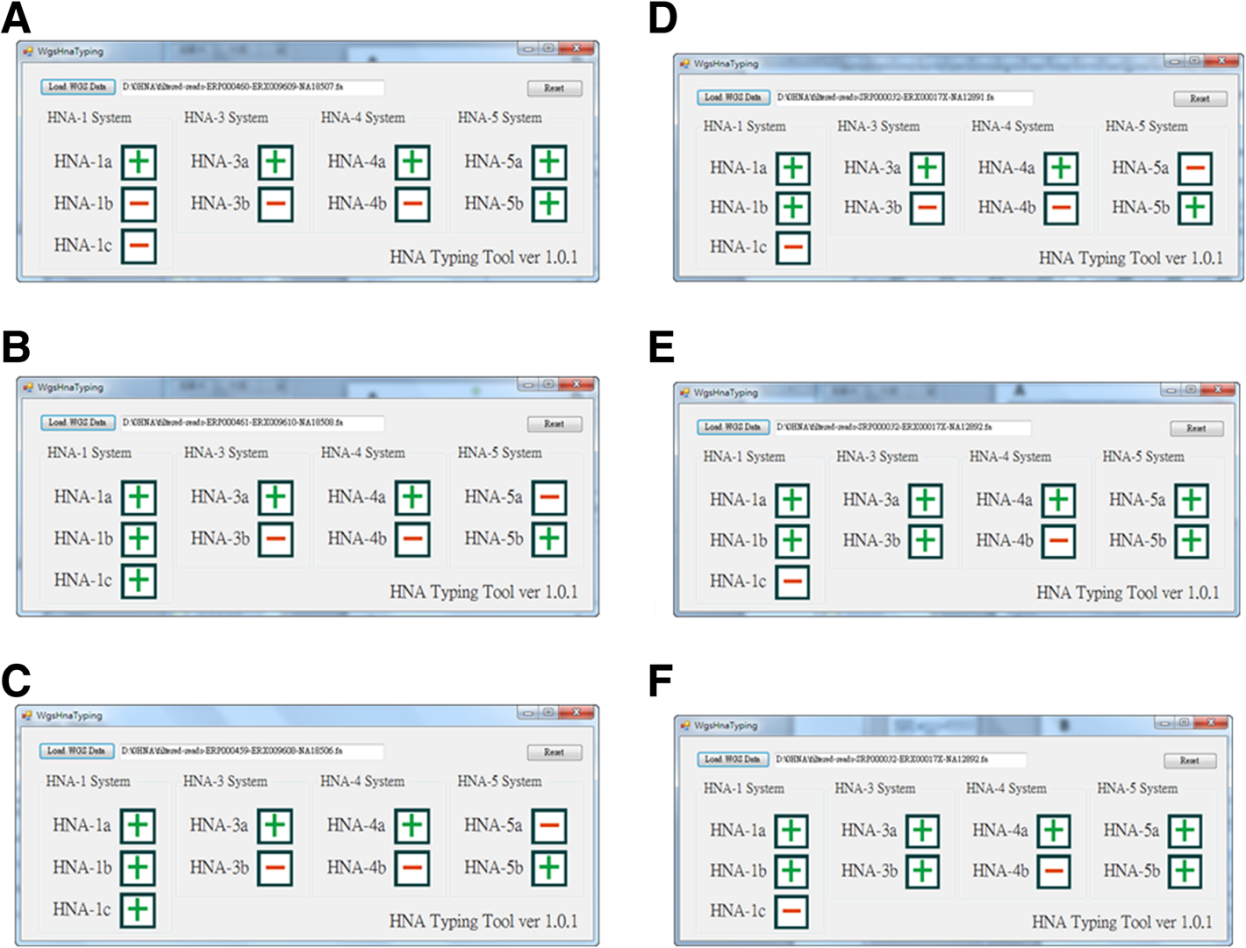
**Results of HNA genotyping for the six samples.** The results **A**, **B**, **C** are for the pedigree trios YOR009 and the others are for the pedigree trios YOR009.

**Figure 4:**
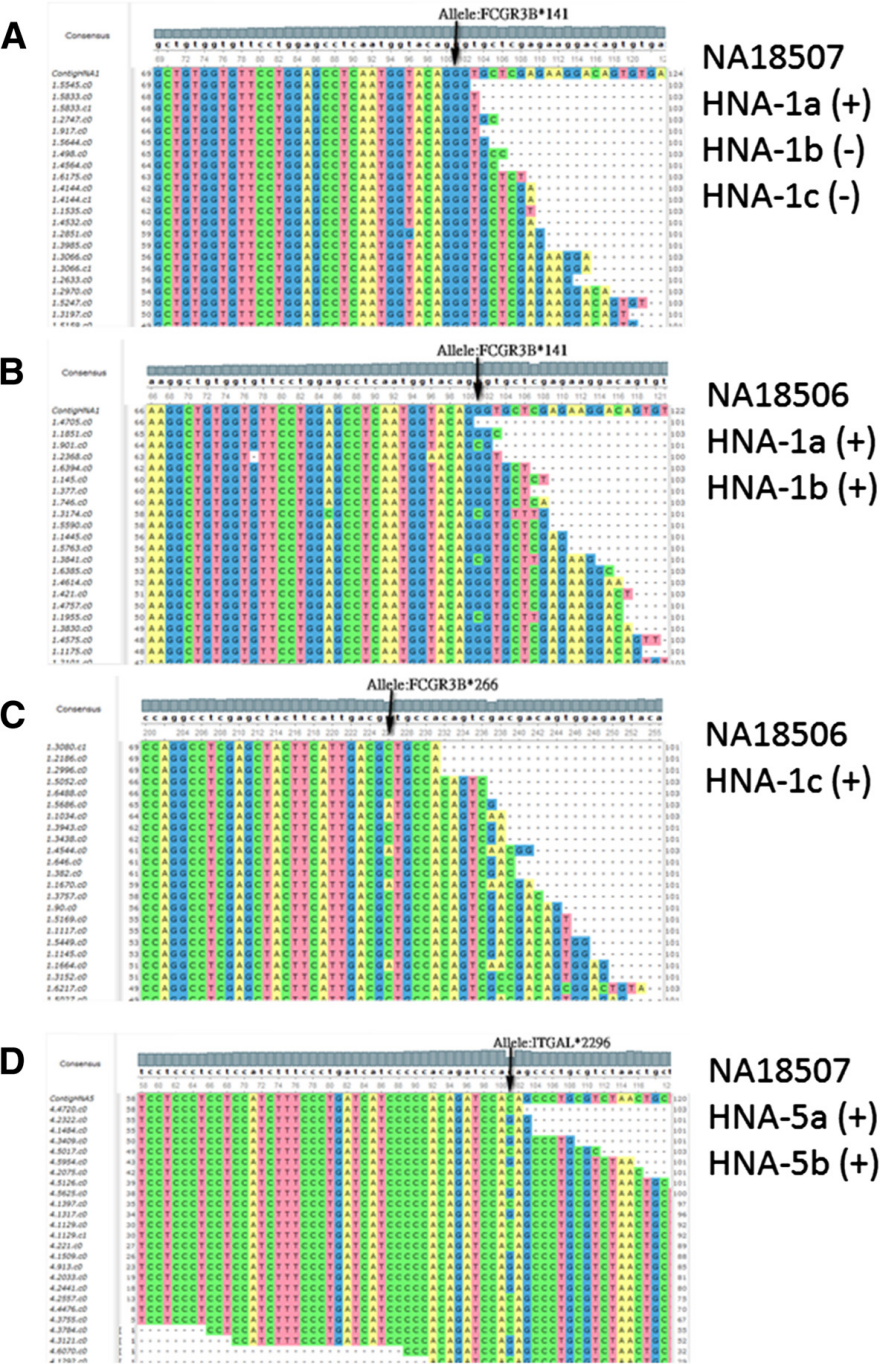
**Read alignment for the significant alleles. A)** The allele:FCGR3B*141G indicates only the genotype HNA-1a in this case. **B)** The alleles:FCGR3B*141G- > C verify the additional HNA-1b/HNA-1c types from HNA-1a. **C)** The allele:FCGR3B*266C- > A determines the existence of HNA-1c. **D)** The allele:ITGAL*2296 shows heterozygous HNA-5 type for the sample NA18507.

The results of HNA typing for the samples are displayed in both of Figures 3 and 4. These figures show that all of the typing screens from the WgsHnaTyping program in Figure 3. We illustrate the alignment results of alleles using the UGENE program in Figure 4 for the verification of typing results.

### Typing result of the HNA1 system

In the results, all of the six samples have HNA-1a antigens and only one African was without HNA-1b. Besides, two African samples have HNA-1c which was a rare type for other populations around the world. Figure 4A depicts the consensus of the allele:FCGR3B*141. All of the aligned nucleotides are Guanine and no Cytosine, thus only the HNA-1a type is positive and both the HNA1b and HNA1c types are negative for the sample NA18507. On the contrary, the same location in Figure 4B shows a different situation that both Guanine and Cytosine coexist for the sample NA18506. Combined with the allele (FCGR3B*266) in Figure 4C, it shows that the HNA-1a, HNA-1b and HNA-1c types are all positive.

The likely coexistence of 3 alleles (especially FCGR3B*266) for the sample NA18506 may need verification studies at aspects including the specific DNA structure for sequencing base preferences [20], the additional HNA loci on the same chromosome due to the unequal crossing-over events [21] and due to the gene duplication events [22], and the heterogeneous neutrophil specimen of mixed clonal lineages. Albeit, the detected coexistence of 3 alleles maybe beneficial for avoiding clinical crisis caused by the false negative results.

### Typing result of the HNA3, HNA4 and HNA5 systems

In the typing of HNA-3, -4, -5 systems, there were four homozygous cases of HNA-3aa and two heterozygous cases of HNA-3ab. Similarly, there were three homozygous cases of HNA-5bb and three heterozygous cases of HNA-5ab as well. Besides, all of the six samples were homozygous in the typing of HNA-4. The results showed that the usefulness of genotyping from whole-genome sequencing. It provided an unambiguous analysis for the zygosity of alleles. Figure 4D illustrates a heterozygous case of HNA-5ab. There were sufficient reads aligned which contribute the nucleotides at the SNP 2296C/G. The SNP confirmed existence of both HNA-5a and HNA-5b antigens in the sample NA18507.

### Requirement of sequencing coverage for HNA-Typing

The genomic data from human whole genome sequencing provided an examination of total variance of personal genome including millions of nucleotide differences. However, the identification of significant SNPs is still a challenging task because the number of revealed alleles is gradually increasing. Moreover, the cost of whole genome sequencing is still expensive so far. As a result, we checked the requirement of sequencing coverage for the HNA alleles. We assumed that the identification of undoubted SNPs required at least two occurrences of each haploid for a total of 4 read alignments. We can use the Poisson distribution to compute the probability of a base being sequenced a certain number of times as:

$$P\left(X=n\right)=\frac{C^{n}\times e^{-c}}{n!}$$

Where n is the number of times a base is read, and C stands for coverage. The coverage of WGS dataset was defined as the total bases from the sequences reads divided by the size of human genome. (The estimated diploid genome sizes for human female and male genomes are 6.406G and 6.294G, respectively [23].)

Therefore, the probability of the base being sequenced less than 2 times is

$$P\left(X\leq 1\right)=P\left(X=1\right)+P\left(X=0\right)$$

Finally, the probability of having at less than two occurrences of each haploid can be obtained by calculating *P(X*_*1*_*≤ 1 ∪ X*_*2*_*≤ 1)*. For example, a coverage of 8X will result in a probability of 99.4% for the event which each haploid at a particular locus is sequence at least twice. Table 4 lists the found nucleotides for the critical alleles of HNA antigens and the sequencing coverage. Table 5 lists the probabilities for the event which each haploid at a particular locus is sequence at least twice for coverages 5 through 12.

**Table 4:** Counts for the critical alleles of HNA antigens

**HapMap family**	**Sample**	**HNA-1**^**a**^**C|A**	**HNA-3**^**b**^**A|G**	**HNA-4**^**c**^**A|G**	**HNA-5**^**d**^**C|G**	**Coverage**
YOR009	NA18507	1|87	0|21	0|21	14|12	21.29
	NA18508	20|81	0|36	0|23	0|29	20.71
	NA18506	27|90	0|30	0|41	0|45	20.97
CEPH146	NA12891	0|21	0|2	0|8	0|7	5.61
	NA12892	0|8	3|2	0|5	4|1	5.50
	NA12878	0|23	3|1	0|7	3|6	6.23

^a^The nucleotides at the locus of allele FCGR3B*266.

^b^The nucleotides at the locus of allele CTL2*461.

^c^The nucleotides at the locus of allele ITGAM*230.

^d^The nucleotides at the locus of allele ITGAL*2372.

**Table 5:** Probabilities for the event which each haploid at a particular locus is sequence at least twice

**Coverage**	**P(X1 > 1∩ *X *2 > 1)**
5	92.078%
6	96.560%
7	98.546%
8	99.397%
9	99.753%
10	99.900%
11	99.960%
12	99.984%
13	99.994%
14	99.998%
15	99.999%

## Conclusions

Our study provides a new approach of genotyping to the HNA systems. The conventional samples, analysed by PCR-based methods, cannot be easily used for testing new variants. However, once there are any new variant such as HNA-1d of the antigen systems discovered and confirmed [21], our bioinformatics tool can be easily modified to explore the WGS datasets again to find the updated and/or undiscovered variants. Our tool may adequately exploit the genotyping advantage of next generation sequencing data either for the HNA systems or for the extended systems such as human leukocyte antigen (HLA) systems.

## Availability and requirements

In the HnaTyping software package, both the programs WgsReadFilter and WgsHnaTyping were implemented in C# with the .NET Framework which can be run on 64-bit Windows/Linux. The HnaTyping software with a user manual is available at the Web site: http://sourceforge.net/projects/hnatyping/.

## Competing interests

The authors declare that they have no competing interests.

## Authors’ contributions

HTC devised the method and wrote the software. HTC, HL, TTHT, CFC, WWLH, TJY, CMC, YWL, TYW, KCY, TJC, JCC and KCC discussed the project and jointly wrote the manuscript. CYK leads the project. All authors read and approved the final manuscript.

## Pre-publication history

The pre-publication history for this paper can be accessed here:

http://www.biomedcentral.com/1755-8794/6/31/prepub
